# A Surveillance System to Reduce Transmission of Pandemic H1N1 (2009) Influenza in a 2600-Bed Medical Center

**DOI:** 10.1371/journal.pone.0032731

**Published:** 2012-03-13

**Authors:** Tsui-Ping Chu, Chung-Chen Li, Lin Wang, Li-Wen Hsu, Hock-Liew Eng, Huey-Ling You, Jien-Wei Liu, Chi-Chen Wei, Ling-Sai Chang, Ing-Kit Lee, Kuender D. Yang

**Affiliations:** 1 Department of Nursing, Chang Gung Memorial Hospital, Chiayi, Taiwan; 2 Department of Pediatrics, Kaohsiung Chang Gung Memorial Hospital and Chang Gung University College of Medicine (KCGMH-CGU), Kaohsiung, Taiwan; 3 Department of Pathology, KCGMH-CGU, Kaohsiung, Taiwan; 4 Division of Infectious Diseases, Department of Internal Medicine, KCGMH-CGU, Kaohsiung, Taiwan; 5 Department of Medical Research, Show Chwan Memorial Hospital in Chang Bing, Changhua, Taiwan; 6 Department of Pediatrics, Show Chwan Memorial Hospital in Chang Bing, Changhua, Taiwan; Tulane School of Public Health and Tropical Medicine, United States of America

## Abstract

**Background:**

Concerns have been raised about how the transmission of emerging infectious diseases from patients to healthcare workers (HCWs) and vice versa could be recognized and prevented in a timely manner. An effective strategy to block transmission of pandemic H1N1 (2009) influenza in HCWs is important.

**Methodology/Principal Findings:**

An infection control program was implemented to survey and prevent nosocomial outbreaks of H1N1 (2009) influenza at a 2,600-bed, tertiary-care academic hospital. In total, 4,963 employees at Kaohsiung Chang Gung Memorial Hospital recorded their temperature and received online education on control practices for influenza infections. Administration records provided vaccination records and occupational characteristics of all HCWs. Early recognition of a pandemic H1N1 (2009) influenza case was followed by a semi-structured questionnaire to analyze possible routes of patient contact, household contact, or unspecified contact. Surveillance spanned August 1, 2009 to January 31, 2010; 51 HCWs were confirmed to have novel H1N1 (2009) influenza by quantitative real-time reverse transcription polymerase chain reaction. Prevalence of patient contact, household contact, or unspecified contact infection was 13.7% (7/51), 13.7% (7/51), and 72.5% (37/51), respectively. The prevalence of the novel H1N1 infection was significantly lower among vaccinated HCWs than among unvaccinated HCWs (p<0.001). Higher viral loads in throat swabs were found in HCWs with patient and household contact infection than in those with unspecified contact infection (4.15 vs. 3.53 copies/mL, log_10_, p = 0.035).

**Conclusion:**

A surveillance system with daily temperature recordings and online education for HCWs is important for a low attack rate of H1N1 (2009) influenza transmission before H1N1 (2009) influenza vaccination is available, and the attack rate is further decreased after mass vaccination. Unspecified contact infection rates were significantly higher than that of patient contact and household contact infection, highlighting the need for public education of influenza transmission in addition to hospital infection control.

## Introduction

H1N1 (2009) influenza was another pandemic infection that followed the severe acute respiratory syndrome (SARS) pandemic. During the 2003 SARS outbreak, our 2600-bed medical center experienced a serious nosocomial infection that resulted in closure of the facility in a bid to contain the hospital-acquired infection [1]. Since then, concerns have been raised about how the transmission of emerging infectious diseases from patients to healthcare workers (HCWs) and vice versa could be recognized and prevented in a timely manner [2], [3]. Thus, the hospital infection control team has been enhancing infection control training for HCWs and implementing hospital-wide surveillance during outbreaks of infection.

The pandemic H1N1 (2009) influenza outbreak, which emerged from the United States and Mexico in April 2009, rapidly spread worldwide, and cases were recorded even in the winter of 2010 [4], [5]. The first imported case in Taiwan was diagnosed in May 2009 [6] and the virus spread to the community in July the same year [7]. After this development, our infection control team immediately designed a series of strategies to enhance our colleagues' awareness and prevention of hospital-acquired infections. During an influenza pandemic, the major concerns are that the increased demand for healthcare service, particularly emergency rooms and inpatient beds, and increased absenteeism of sick HCWs will reduce the quality of healthcare provided.

There are a number of reports concerning the incidence, transmission, and risk factors for H1N1 influenza infection in HCWs [8], [9], [10]. Thus, an effective strategy to block transmission of pandemic H1N1 (2009) influenza in HCWs is important. This study was conducted to investigate the different transmission routes among HCWs and risk factors for infection among different subgroups of HCWs. Analysis was also carried out to determine whether infection control practices and vaccination protected HCWs from novel H1N1 (2009) influenza infection.

## Methods

### Ethics statement and participants

This study was approved by the Institution Review Board (IRB) of Kaohsiung Chang Gung Memorial Hospital (KCGMH), Taiwan. Our study was conducted at KCGMH, a 2600-bed tertiary-care center in southern Taiwan. According to the KCGMH annual census reports, 3986 of the 4963 HCWs who worked for KCGMH in 2009 were involved in direct patient care. We obtained informed consent of H1N1-infected HCWs to collect the semi-structured questionnaires with personal information, history and data protected by infection control team.

### Case definition

HCWs were defined as employees of KCGMH providing clinical or nonclinical services. The clinical HCW group included those involved in patient care, such as physicians, nurses, nursing assistants, therapists, pharmacists, infection control nurses, and laboratory personnel. Nonclinical HCWs were defined as those who were not involved in patient care, such as clerical, dietary, housekeeping, and administrative staff, and volunteers. HCWs with H1N1 influenza infection were defined as those who had influenza-like illness (ILI) and were positive for H1N1 influenza by real-time reverse transcription polymerase chain reaction (RT-PCR). ILI was defined as fever (body temperature ≧38°C), cough, and/or sore throat in the absence of a known cause other than influenza. Patient contact infection was defined as a laboratory-confirmed case of influenza in a HCW who had contact with a H1N1 influenza-confirmed patient during his/her course of work in the hospital. Household contact infection was defined as a laboratory-confirmed case of influenza in a HCW who had contact with a H1N1 influenza-confirmed household member at home. Unspecified contact infection was defined as a laboratory-confirmed case of influenza in a HCW who did not belong to the patient contact or household contact infection groups.

### Study design and data collection

**Figure 1:**
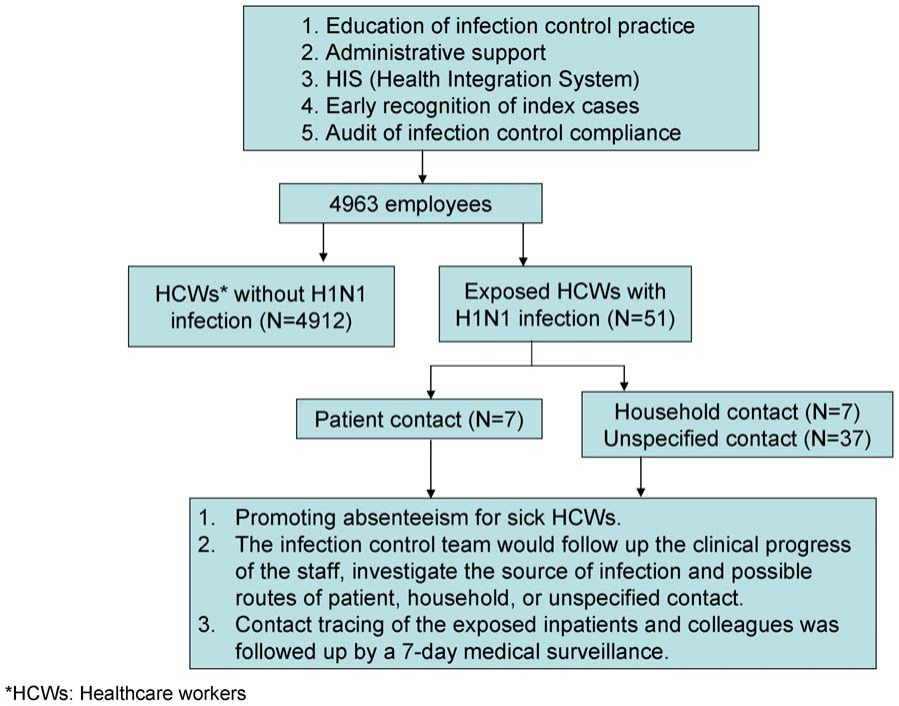
Infection control strategy outcomes for prevention of nosocomial transmission of H1N1 influenza at KCGMH.

The aim of this study was to describe an infection control program implemented to reduce transmission of pandemic H1N1 (2009) influenza in a tertiary-care center (KCGMH) by using an online health integration system (HIS) from August 1, 2009 to January 31, 2010. The infection control program at KCGMH included online education about infection control practices for HCWs, early recognition of index cases in the hospital, and audits for infection control compliance such as surgical mask practice and administrative support, which was modified from the infection control guidelines issued by Centers for Disease Control, Taiwan (Figure 1).

The details of the infection control program were established as listed in Table 1. The online education module offered lectures aimed at improving understanding of not only influenza, but also of emerging or novel diseases, as well as presenting the general principles of infection control and infectious disease prevention, and video or graphic demonstrations of hand washing and gowning/degowning of personal protective equipment. After viewing the lectures, all staff was required to take an online test that consisted of 10 multiple-choice questions. One point was awarded for each correct answer, and a pass was based on 10 correct answers. Staff members who were unable to achieve this goal were required to retake the test with 10 different questions.

**Table 1:** Infection control program in prevention of transmission of pandemic H1N1 (2009) influenza.

**1.**	**Education of infection control practice**
(1)	Traditional and online learning and review of general principles of infection control for all healthcare workers immediately before outbreak of pandemic H1N1 (2009) influenza in community.
(2)	Specific infection control program designed for staff caring for patients with pandemic H1N1 (2009) influenza in isolation rooms.
**2.**	**Administrative support**
(1)	Providing timely supply of manpower and equipment for rapid diagnosis of pandemic H1N1 (2009) influenza.
(2)	Establishing dedicated clinics for patients with flu-like symptoms to avoid transmission of pandemic H1N1 (2009) influenza from sufferers to non-sufferers crowded at outpatient clinics and in emergency departments in particular.
(3)	Provide 75% alcohol-based solution for hand rub in all inpatient rooms, corridors, and outpatient clinics.
**3.**	**HIS (Health Integration System)**
(1)	Daily online reporting of healthcare workers' body temperatures.
(2)	Information of any febrile healthcare worker or from outpatient clinics routinely reported on daily basis transmitted to an infection control team for further analysis and investigation as necessary.
(3)	Analysis of epidemiologic trends of patients and hospital workers reported to suffer influenza-like illness.
(4)	Immediately alerting the hospital infection control team to any positive laboratory result confirming a newly diagnosed pandemic H1N1 (2009) influenza case.
**4.**	**Early recognition of index cases**
(1)	Screening and triage of symptomatic patients in influenza-like clinic and emergency room, and admission to isolation rooms.
(2)	Prompt referral of the patients with nosocomial onset of respiratory symptoms to isolation rooms.
(3)	Availability of real-time reverse transcription polymerase chain reaction (RT-PCR) results of clinical specimens from patients with clinically suspected pandemic H1N1 (2009) influenza within 24 h.
**5.**	**Audit of infection control compliance**
(1)	Ensuring adherence to strict hand hygiene and surgical mask wearing under supervision of infection control practitioners.
(2)	A designed questionnaire dispatched to assess if the healthcare workers understand the safe infection control practice.
(3)	Assessment of the understanding of the infection control practice in isolation rooms among healthcare workers.

The infection control program also required all HCWs to record their daily body temperature in an online reporting system. The temperature record was logged online once daily (at approximately 8 AM). The HCWs on night duty logged their temperature before each shift. The HCWs could not log their temperatures on their days off, but were required to report to their team leader within 24 hours if they had body temperatures ≧38°C. If a HCW's body temperature was ≧38°C, he/she was advised to visit the ILI clinic immediately.

A standard and transmission-based precaution protocol in clinical areas, especially regarding strictly observed hand hygiene, was established before the outbreak. Vaccination records and occupation characteristics for all hospital employees were obtained from administrative records. According to infection control guidelines at KCGMH, any HCW who developed ILI should report to the infection control team within 24 hours. Afebrile HCWs suspected of having H1N1 infection were also required to report to the infection control team within 24 hours. Throat (tonsillopharyngeal) swabs from sick HCWs were obtained for diagnosis of H1N1 influenza by a quantitative RT-PCR assay developed in our previous study [11]. After diagnosis was confirmed, a member of the infection control team conducted a face-to-face interview with the HCW to complete a semi-structured questionnaire to analyze possible routes of patient, household, or unspecified contact. The content of the questionnaire included the HCW's age, sex, symptoms/signs, onset of disease, coexisting condition(s), working location, and TOCC (travel, occupation, contact, cluster) history. HCWs confirmed with H1N1 infection received a 7-day off clinical duty period. All of the strategies were implemented at the same time and were initiated after the first imported case was diagnosed in Taiwan while there was no case of ILI among HCWs in the hospital.

### Statistical methods

Demographic characteristics and possible transmission routes are presented as numbers (percentages). Viral load for RT-PCR are presented as mean ± SEM values. Differences among groups regarding to occupation, working location and vaccination were determined by using Fisher exact test or univariate logistic regression. Odds ratio (OR) values were calculated with 95% confidence intervals (CI). Student's *t*-test was used for statistical comparisons between continuous variables. P value of less than 0.05 was statistically significant. All analyses were performed using SPSS 13.0 (SPSS Inc., Chicago, IL, USA).

## Results

### Infection control preparedness for pandemic H1N1 (2009) influenza

Between May 4 and 16, 2009, 14 lecture sessions with an overall attendance of 4689 (94.5%) hospital staff were held to provide up-to-date knowledge and infection control practice to prevent transmission of H1N1 influenza. Online training was held twice before the outbreak of H1N1 influenza in the community. The rate of HCWs who attended each online training session was 91.8% in May and 97.3% in July. Each person required at least 1 hour to complete each session, and were required to pass by taking an online test that comprised 10 questions. The vaccination program was not included in the original infection control strategy, but was introduced on November 1, 2009, according to the national vaccination policy. For the monthly audit of hand hygiene practice observed by the members of infection control team, the overall compliance rate of HCWs ranged 88.2–95.7% throughout the duration of surveillance. The internal medicine ward had the lowest compliance rate (84.2%) in August. The audit of gowning practices of personal protective equipment in isolation rooms was respectively performed in August, October, and December 2009, the overall compliance rate reached 100% consistently.

The daily rate of HCWs who filled out the body temperature log ranged 95.8–96.4%. One hundred and seventy-five HCWs reported fever. Of those febrile HCWs, 37 (21.1%) had H1N1. There was no significant difference in age, sex distribution, and working location between the non-H1N1 HCWs and H1N1 HCWs. Fourteen afebrile HCWs with respiratory tract symptoms (cough, rhinorrhea or sore throat) were reported to the infection control team via HIS after they visited the outpatient clinic and were laboratory-confirmed with H1N1 infection. Of the 138 febrile but non-H1N1 HCWs, 105 had upper respiratory tract infection caused by viruses other than influenza, 15 had gastrointestinal tract infection caused by viruses other than influenza, 8 had lower respiratory tract infection caused by bacteria, 7 had urinary tract infection, and 3 had cellulitis.

### Pandemic H1N1 (2009) influenza in the hospital

**Figure 2:**
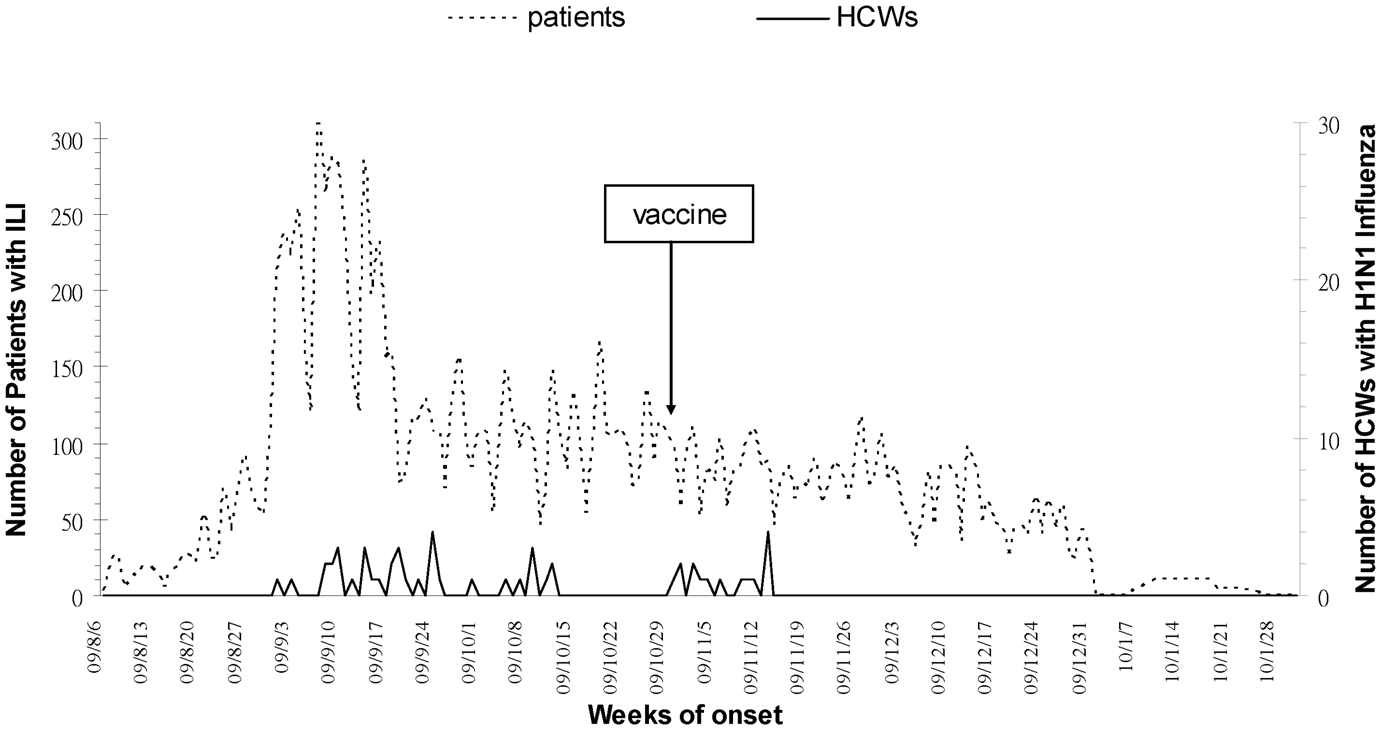
Distribution of patients with ILI and HCWs with H1N1 influenza from August 1, 2009 to January 31, 2010.

Throughout the 6-month surveillance period, the number of patients with ILI who visited our hospital for treatment peaked in early September and declined significantly after a national vaccination program was initiated in Taiwan on November 1, 2009 (Figure 2). HCWs reporting H1N1 infection occurred most frequently in September, which was compatible with the peak number of patients with ILI in early September (Figure 2). Similarly, the number of HCWs with H1N1 infection declined after national vaccination was introduced. There were no fatal cases in the cohort analysis.

### Patient, household, or unspecified contact with H1N1 (2009) influenza

Throughout the duration of surveillance, 51 HCWs (1.0%) were confirmed to have H1N1 infection (Table 2). These included 47 clinical HCWs (92.2%) and 4 nonclinical HCWs (7.8%). The attack rate was significantly higher in clinical HCWs than in nonclinical HCWs (1.2% vs. 0.4%; p = 0.032). The OR was 2.90 (95% CI: 1.04–8.08). The mean age of the 51 HCWs was 31.9±6.6 years (range, 19–48 years). The majority of the HCWs were women (84.3%). Unspecified contact infections (37/51) were the most frequently reported infection/exposure routes, followed by patient contact (7/51) and household contact infections (7/51). The percentage of clinical HCWs reporting unspecified contact infection was higher than the percentage of those infected by other routes of transmission. Among the sick HCWs, 8 had a coexisting condition; 5 had hypertension (9.8%), 2 had asthma (3.9%), and 1 had nephrotic syndrome (2.0%).

**Table 2:** Characteristics of healthcare workers (HCWs) with H1N1 influenza infection.

Variables	Clinical HCWs (%)N = 47	Nonclinical HCWs (%)N = 4
Age groups (years)		
<30	18 (38.3)	1 (25.0)
31–40	22 (46.8)	2 (50.0)
41–49	7 (14.9)	1 (25.0)
Gender		
Male	6 (12.8)	2 (50.0)
Female	41 (87.2)	2 (50.0)
Exposure		
Patient contact	7 (14.9)	0 (0)
Household contact	7 (14.9)	0 (0)
Unspecified contact	33 (70.2)	4 (100)
Coexisting condition		
None	39 (83.0)	4 (100)
Asthma	2 (4.3)	0 (0)
Hypertension	5 (10.6)	0 (0)
Nephrotic syndrome	1 (2.1)	0 (0)

### Professional association of pandemic H1N1 (2009) influenza

The distribution of attack rate for H1N1 influenza among HCWs is presented in Table 3. The attack rate among different HCW groups was significantly different (p = 0.027). The attack rate was highest among nurses (1.5%), followed by physicians (1.0%), other clinical HCWs (0.7%), and administrative and ancillary workers (0.4%). The distribution of infection based on work location was significantly different (p<0.001). The attack rate was highest among wards (2.1%), followed by outpatient departments (1.9%), emergency rooms (1.7%), intensive care units (1.2%), and was lowest in non-patient care locations (0.2%). Furthermore, 47 HCWs (0.9%) were infected with H1N1 influenza before the vaccination was introduced. The other 4 HCWs were infected with H1N1 influenza even after they had received vaccination. It was clear that the HCWs without vaccination experienced a significantly higher attack rate than did those with vaccination (p<0.001) during the surveillance period.

**Table 3:** Attack rates for healthcare workers (HCWs) with H1N1 influenza infection classified by occupation, work location, and vaccination status.

Variables	Number of participants	Number of infections (%)	OR (95% CI)	p value
Occupation				**0.027**
Nurse	2032	31 (1.5)	1	
Physician	778	8 (1.0)	0.67 (0.31–1.47)	0.316
Other clinical HCW	1176	8 (0.7)	0.44 (0.20–0.97)	0.040
Administrative and ancillary worker	977	4 (0.4)	0.27 (0.09–0.75)	0.013
Working location				**<0.001**
Wards	1201	25 (2.1)	1	
OPD	639	12 (1.9)	0.90 (0.45–1.80)	0.767
ER	172	3 (1.7)	0.84 (0.25–2.80)	0.770
ICU	577	7 (1.2)	0.58 (0.25–1.34)	0.203
Non-patient care locations	2374	4 (0.2)	0.08 (0.03–0.23)	<0.001
Vaccination				
Pre-vaccination	4963	47 (0.9)	1	**<0.001**
Post-vaccination	4740	4 (0.1)	0.09 (0.03–0.25)	

ER, emergency room; ICU, intensive care unit; OPD, outpatient department; OR, odds ratio; CI, confidence interval.

**Figure 3:**
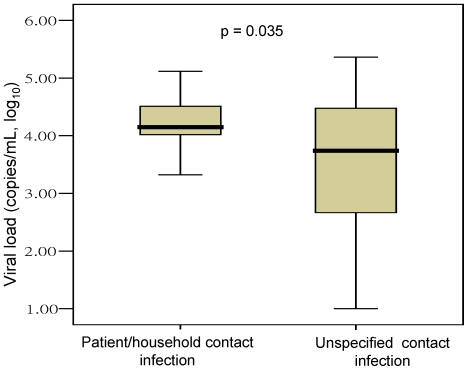
Comparison of viral loads in throat swabs from HCWs with different transmission routes.

Cough (90.5%), fever exceeding 38°C (72.5%), rhinorrhea (68.2%), sore throat (56.7%) and muscle aches (21.6%) were the most frequently reported symptoms among HCWs with H1N1 influenza infection. Through analysis of the correlation between viral loads and different routes of transmission, we found that the viral load was significantly higher in HCWs with patient contact and household contact infections than in those with unspecified contact infections (4.15 vs. 3.53 copies/mL, log_10_, p = 0.035) (Figure 3).

## Discussion

In this study, we described the surveillance for H1N1 influenza among HCWs in a large medical facility. During the 6-month surveillance, 51 (1.0%) HCWs were confirmed to have H1N1 influenza infection. The attack rate among HCWs was 0.9% before the vaccination program was implemented, and was lower than that in another study (5.4%) [10]. It means that the infection control program in this study was observed to have a low attack rate of confirmed H1N1 cases. The low attack rate was attributable to an intensive infection control/monitoring/education program and universal vaccination policy. More importantly, we found that the number of infections acquired from patient care was much lower than that acquired from unspecified contact in the 2600-bed hospital cohort. This finding suggests that infection control is the most important strategy for control of H1N1 influenza transmission in a hospital before the availability of H1N1 influenza vaccination, and although HCWs may take every precaution to guard against acquiring influenza from patient care, they may still be infected from non-patient care sources.

The KCGMH guidelines for H1N1 influenza were adapted from the experience with the SARS outbreak and were approved by a multidisciplinary team led by the infection control department. These guidelines were locally customized according to the guidelines defined by the World Health Organization and Centers for Disease Control and Prevention (CDC) [12], [13]. The low attack rate for hospital-acquired infection indicated that the infection control program was effective. Particularly, HCWs with H1N1 infection under medical surveillance were advised to be released from clinical duty and required to seek medical support for appropriate treatment. This strategy could minimize the risk of transmission among HCWs and patients in the hospital. Our study showed that an online HCW surveillance system is necessary for the detection of hospital transmission of pathogens, including novel H1N1 (2009) influenza virus. Implementation of hospital-wide surveillance, early detection, and early antiviral use will be important to limit the transmission of an emerging infectious disease such as H1N1 influenza among HCWs.

The overall 6-month attack rate was 1.0% among HCWs. As expected, the risk was not homogenous throughout the hospital. This study also found that nurses were at the highest risk for infection among all HCW subgroups; this finding is similar to that of other studies [8], [10]. Regarding work location, emergency rooms and pandemic (H1N1) isolation wards have been reported to be places with the highest risk [8], [10]. However, our study found that ordinary wards were the work locations with the highest risk. The reason that nurses were at a higher risk remains unclear; a possible explanation is that they were exposed more frequently to sick patients and the patients' relatives and visitors than were other HCWs during their course of work. The wards experienced the highest attack rate, probably because the HCWs in those wards had less stringent protection measures than other HCWs did.

In addition to exposure to infected patients, contact with sick colleagues and household members were reported to be associated with HCWs infected with H1N1 influenza [8], [10]. However, the highest rate of exposure for HCWs in this study was due to unspecified contact. HCWs who acquired H1N1 influenza appeared to have been infected from non-patient care exposure or in social settings with colleagues. This phenomenon highlights the necessity of public education to prevent further influenza transmission. In addition, the viral loads in throat swabs were higher in HCWs with patient contact and household contact infections than in those with unspecified contact infections. A possible explanation is that the HCWs in the first group were infected with H1N1 influenza via direct, close contact with patients in confined spaces such as wards or homes.

The influenza vaccine is effective in reducing mortality and morbidity in children, the elderly, or debilitated patients [14], [15], [16]. It has been shown to prevent infection in HCWs and may reduce the days of absence from work during an influenza epidemic [17]. In this study, the number of patients with H1N1 infection decreased after vaccination was introduced. The infection rate among HCWs with H1N1 infection was significantly lower after vaccination than before vaccination. In addition, the increase in influenza vaccination was associated with a significant decrease in influenza among HCWs [18]. The rate of influenza vaccination in HCWs was 95.5% in the study hospital. This high rate of vaccination exceeded the target rate (80%) recommended by CDC [19], and partly contributed to the low rate of H1N1 infection in HCWs at the hospital.

In conclusion, the surveillance systems, including a hospital control strategy with daily temperature recording and online education about infection control, were observed to have a low attack rate of H1N1 influenza transmission in a 2600-bed hospital before H1N1 (2009) influenza vaccination available, and the attack rate is further decreased after mass vaccination. A further seroepidemiologic investigation might be needed to detect the HCWs with H1N1 who were not identified by the infection control strategies. Nurses were at a higher risk of infection than were other HCWs. The number of HCWs with unspecified contact infections was significantly higher than were those with patient contact and household contact infections, highlighting the importance of public education on influenza transmission.
